# Dark septate endophyte improves salt tolerance of native and invasive lineages of *Phragmites australis*

**DOI:** 10.1038/s41396-020-0654-y

**Published:** 2020-04-27

**Authors:** Martina Gonzalez Mateu, Andrew H. Baldwin, Jude E. Maul, Stephanie A. Yarwood

**Affiliations:** 1 Environmental Science and Technology, University of Maryland, College Park, MD 20742 USA; 2 Sustainable Agricultural Systems Laboratory, USDA-Agricultural Research Service, Beltsville, MD 20705 USA

**Keywords:** Freshwater ecology, Plant ecology, Fungal ecology, Microbial ecology, Microbial ecology

## Abstract

Fungal endophytes can improve plant tolerance to abiotic stress. However, the role of these plant–fungal interactions in invasive species ecology and their management implications remain unclear. This study characterized the fungal endophyte communities of native and invasive lineages of *Phragmites australis* and assessed the role of dark septate endophytes (DSE) in salt tolerance of this species. We used Illumina sequencing to characterize root fungal endophytes of contiguous stands of native and invasive *P. australis* along a salinity gradient. DSE colonization was assessed throughout the growing season in the field, and effects of fungal inoculation on salinity tolerance were investigated using laboratory and greenhouse studies. Native and invasive lineages had distinct fungal endophyte communities that shifted across the salinity gradient. DSE colonization was greater in the invasive lineage and increased with salinity. Laboratory studies showed that DSE inoculation increased *P. australis* seedling survival under salt stress; and a greenhouse assay revealed that the invasive lineage had higher aboveground biomass under mesohaline conditions when inoculated with a DSE. We observed that *P. australis* can establish mutualistic associations with DSE when subjected to salt stress. This type of plant–fungal association merits further investigation in integrated management strategies of invasive species and restoration of native *Phragmites*.

## Introduction

Fungal endophytes establish mutualistic associations with most plant species, and can play a major role in plant ecology and community structure [1]. These endophytes can improve host nutrient uptake [2, 3], improve host defense against pathogens [4], modify trophic interactions [5–7], and improve host tolerance to abiotic stress [8, 9]. At the plant community level, they can affect plant diversity [10, 11] and can be important factors in plant invasion ecology [12, 13]. A better understanding of plant–microbe interactions can help improve various aspects of invasive species management [14]. Kowalski et al. [15] recently proposed a framework for a microbial-based control strategy of invasive species; the basis of this strategy is that greater understanding of key microbial association of invasive and native species can lead to new insights of invasive species’ success and improve management practices.

The aggressive expansion of the invasive European lineage of *Phragmites australis* is an issue in several regions of the United States. Management of this lineage has been costly. Despite agencies spending over $4.6 million/year [16], most eradication efforts are unsuccessful and focused on short-term results [15, 17]. Once established, invasive *P. australis* forms dense monotypic stands affecting native plant diversity [18–20], hydrology [21], and biogeochemistry [22, 23] in invaded areas. Expansion of this lineage has been common in brackish marshes [24, 25] and salt marshes, where it can significantly alter ecological functions [19, 26].

The native North American haplotype F of *P. australis* [27] is less salt tolerant than the invasive European haplotype M [28], and is therefore predominantly found in low-salinity habitats [29]. Both lineages share the same physiological mechanisms of salt tolerance, which are K^+^ accumulation in plant tissues and Na+ exclusion [30, 31]; but the growth potential of the invasive lineage has been considered key to its invasiveness at higher salinities [28]. Expansion of the invasive lineage into salt marshes has also been related to clonal integration [32] and temporary decreases in soil salinity [20, 33]. Benefits of microbial associations for salinity tolerance of *P. australis* have been theorized [34] but have not been assessed until this study.

In wetlands, one of the most common groups of root endophytes are dark septate endophytes (DSE). In these systems they are commonly found to coexist with mycorrhizal fungi and are more prevalent in monocotyledonous than dicotyledonous plant species [35, 36]. DSE are considered generalist root fungi and have been found to associate with over 600 plant species, including some that are non-mycorrhizal in various ecosystems [37, 38]. Based on the classification by Rodriguez et al. [1], these Class IV endophytes can be characterized as sterile or conidial, they have dark melanized hyphae and microsclerotia, and are likely to play an important role in plant ecophysiology. Several studies have found DSE colonization is common in plants exposed to abiotic stress [39–41], and experimental inoculation of plants with DSE has been reported to improve host tolerance to heavy metal contamination [42] and drought [43]. Some of the possible mechanisms by which DSE can affect host fitness include the production of bioactive compounds [42, 44], and increasing nutrient uptake by colonized hosts [2, 43, 45]. Considering the ubiquitous nature of DSE in wetland grass species and their ability to promote stress tolerance in various hosts, their associations with wetland plants, and potential functional roles merit further investigation. Specifically, their interactions with native and invasive plants like *P. australis* could be of interest to improve management of the invasive lineage as proposed by Kowalski et al. [15].

In this study we characterized the fungal endophyte communities of contiguous stands of native and invasive *P. australis* across a salinity gradient. We used next generation sequencing and microscopy to address the role of lineage and salinity in structuring root fungal communities over a growing season. In pursuing this objective, we identified salinity-driven DSE colonization patterns that led to a follow-up question: Can fungal endophytes improve salt tolerance of *P. australis*? We hypothesized that DSE mutualists played a role in stress tolerance of the invasive *P. australis* lineage, and used laboratory and greenhouse assays to test this prediction.

## Materials and methods

### Study sites and sampling

**Fig. 1:**
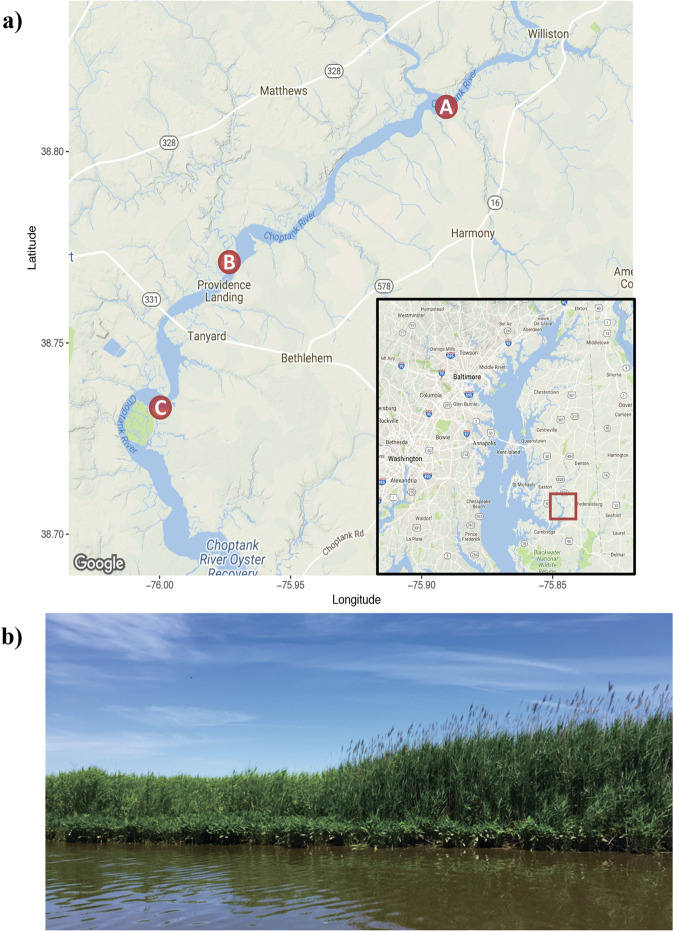
Sampling locations. **a** Sites located along the Choptank River in the Chesapeake Bay following a salinity gradient over 12.5 km (Site A = ~0.7 ppt, Site B = ~1.2 ppt, and Site C = ~3 ppt). **b** Example of contiguous stand of native (left, shorter) and invasive (right, taller) *P. australis* in Site B.

We selected three sites with contiguous stands of native and invasive *P. australis* along a salinity gradient in the Choptank River in eastern Maryland, USA (Fig. 1). The salinity regimes at these tidal wetland sites range from freshwater (<0.5%) to oligohaline (0.5–5%) [46]. During the summer of 2016, we collected rhizomes from visibly healthy native and invasive *P. australis* by excavating the plant and clipping rhizomes that had multiple lateral and fine roots. Four rhizomes were sampled from each stand from plants that were at least 5 m away from each other. Sampling was carried out approximately every 2 weeks between June and October, resulting in a total of 84 rhizomes of each lineage that were collected for analysis (168 total rhizomes-3 sites × 2 lineages × 7 time points × 4 rhizomes/plant). To monitor water level, we installed loggers (HOBO U20L-04, Bourne MA, USA) in stands of native and invasive *P. australis* at sites A and C, and water level was recorded every 5 min from July to October. We calculated the level of inundation for each stand based on the percent time that the water was above the soil surface over the 2-week period before each rhizome sampling date. Salinity was recorded at each site using a portable salinity meter (YSI, Yellow Springs OH, USA). We collected six soil samples at a depth of 25 cm from each site in July and analyzed their pH using a 1:5 soil:DI water slurry, soil organic matter (SOM) using loss-on-ignition (550 °C for 2 h) and then sent them to the Delaware Soil Testing Lab for analyses of percent nitrogen (%N) and carbon (%C) by combustion at 950 °C. We characterized root morphology of native and invasive *P. australis* based on three samples from each stand and measured lateral root density and length, and root hair density [47].

### Root processing and endophyte sequencing

Lateral and fine roots were clipped and separated for different uses. Some were stored in 50% ethanol for staining and microscopy, and the rest were surfaced sterilized and either stored at −80 °C for Illumina sequencing, or used to isolate fungal endophytes. Surface sterilization was carried out as described in Ban et al. [48], with 99% ethanol for 1 m, 35% hydrogen peroxide for 5 m, 99% ethanol for 30 s, and washing three times in sterile DI water. We confirmed the success of the root sterilization by imprinting the roots on potato dextrose agar (PDA) and confirming no signs of growth after incubation.

To isolate root endophytes we clipped the ends of the roots and placed them on PDA with ampicillin (50 μg ml^−1^) and streptomycin (25 μg ml^−1^). Plates were incubated in the dark at 23 °C and after about 10 days fungi that emerged from the roots were transferred to new PDA plates. We characterized the isolated endophytes using Sanger sequencing. Endophyte recovery from the Choptank sites was low and we were only able to obtain one DSE from native and invasive *Phragmites* at Site B and one from Site G after plating four segments of eight root samples collected at each site. Therefore additional isolates were obtained using the same methods from ten root samples of invasive *Phragmites* that were located at a mesohaline site (8 ppt) on the Patuxent River (N38°32′20″, W76°40′3″). We extracted fungal DNA using a Zymo Quick DNA Fungal/Bacterial kit according to the manufacturer’s instructions. BigDye® Terminator v3.1 (ThermoFisher) was used for PCR amplification using the ITS1F/ITS4R and the EF1-728F/EF1-986R primer sets to amplify the internal transcribed spacer (ITS) region and alpha elongation factor (EF), respectively. EdgeBio cleanup plates were used to recover the cleaned sample, which was then vacuum concentrated using a speedvac, resuspended in 20 µl of HiDi formamide, and denatured for 2 m at 95 °C. We processed the resulting sequences using SeqScanner v.1.0 (ABI) to check quality, DNAStar to assemble contigs at 97% similarity, and BLAST (NCBI) to assign taxonomy. Sequences of the isolated endophytes were deposited in GenBank under accession numbers MT094851-MT094863.

Surface sterilized roots from two of our sampling dates (June 30th and August 24th) were used for DNA extractions and subsequent Illumina sequencing of the ITS1 region. We used a PowerPlant Pro DNA isolation kit (MoBio, Carlsbad CA, USA) for DNA extractions and followed the manufacturer’s instructions, except for the lysing step, which was carried out using a FastPrep®-24 (two 60 s cycles at 6 m s^−1^; MP Biomedicals, LLC, Solon OH, USA). We quantified the extracted DNA using a Qubit 2.0 fluorometer and diluted it to 5 ng μl^−1^ for PCR and amplicon sequencing. The ITS region was targeted using the primer + adapter for ITS1F (5′-TCG TCGGCAGCGTCAGATGTGTATAAGAGACAGCTTGGTCATTTAGAGGAAGTAA-3′) and ITS2 (5′-GTCTCGTGGGCTCGGAGATGTGTATAAGAGACAGGCTGCGTTCTTCAT CGATGC-3′) with Illumina adapters. The PCR reaction had 3.5 μl of DNA, 17.5 μl of ThermoScientific^TM^ Phusion^TM^ Flash High-Fidelity PCR Mastermix (Thermo Fisher Scientific), and 7 μl of each primer (1 ng/μl). PCR products were purified using AMPure XP beads (Beckman Coulter, Pasadena CA, USA) following the Illumina protocol (Part # 15044223 Rev. B, support.illumina.com), and indexed using the Illumina Nextera XT 96 index kit. Samples were pooled, and amplicon size of the library was checked using a Bioanalyzer 2100 (Agilent Technologies). We quantified the library using Q-PCR, and the final library was diluted to 12 pM, spiked with 30% PhiX (Illumina), and ran on an Illumina MiSeq using a 600-cycle v3 cartridge. Resulting sequences were deposited in the NCBI Sequence Read Archive (accession numbers: SAMN14121326-SAMN14121358)

Roots that were stored in 50% ethanol were used for fungal colonization assessment using microscopy. They were cleared by autoclaving in 10% KOH for 15 m, acidified in 1% HCl for 20 m, and stained with 0.05% trypan blue for 2 h to detect if arbuscular mycorrhizas were present together with DSE [49]. Roots were destained overnight in 50% glycerol and stored in lactoglycerol. Percent colonization of DSE was assessed by quantifying melanized DSE hyphae and microsclerotia using the grid intercept method [50], with 100 intersections per slide.

### Laboratory and greenhouse experiments

We assessed the salt resistance of the isolated endophytes from the mesohaline site by growing them in PDA with 200, 400, and 600 mM NaCl. We then used all salt tolerant endophytes in a laboratory experiment to evaluate their effect on survival of *P. australis* under salt stress. Seeds were sterilized with 70% ethanol for 2 m, 10% bleach for 5 m, and three sterile DI water rinses.

For the laboratory experiment, we germinated surface sterilized seeds of invasive *P. australis* on 1% agar. After germination, four seedlings were transferred to Magenta boxes containing solid Murashige and Skoog media with 100 mM NaCl. We then added either a disc of actively growing fungi, or a sterile PDA plug as a control next to each seedling. For the less salt-resistant native *P. australis* lineage, we inoculated the seeds prior to adding them to the Magenta Box because 100 mM of NaCl can induce a stress response for this lineage [28] and we speculated that the endophytes might improve host’s chances of surviving the transplant. We added the sterilized seeds to PDA plates with and without endophytes (Control) and after 24 h transferred them to 1% agar for germination. We then added the seedlings to Magenta boxes containing solid MS agar with 100 mM NaCl and ampicillin. We recorded the number of surviving seedlings of native and invasive *P. australis* after 2 months. Based on these results, we selected one of the endophytes for a greenhouse experiment to further evaluate its effect on salinity tolerance of invasive *Phragmites*. In addition, we stained a subset of the seedling roots (as described in the previous section), to confirm fungal colonization by DSE.

For the greenhouse experiment, sterile seeds of invasive *P. australis* were germinated on 1% agar at 14 h of light and a 30/18 °C diurnal temperature shift. We transferred seedlings into Magenta boxes with half-strength MS basal salt agar and ampicillin. After 3 weeks, we planted 23 seedlings into 2L pots containing a sterile mix of 2:1 Sungro potting soil and sand. Plant height was recorded at the beginning of the experiment and used as a covariate for analysis. One week after planting, we began the endophyte treatment by adding a disc of the selected fungal endophyte that was actively growing on PDA near the base of each plant, or a disc of sterile PDA media for control plants. A week later, we began the salt treatments by adding 100 mM of NaCl to irrigation water and gradually increasing additions by 100 mM weekly until the final treatment levels (Mesohaline = 200 mM and Polyhaline = 400 mM) were reached. This gave us a factorial design with three levels of salinity (Freshwater, Mesohaline, and Polyhaline) and two levels of endophyte (Endophyte and No Endophyte). We placed the pots into aluminum pans to collect drainage water, and plants were watered twice weekly with 1 L of tap water. We added NaCl weekly to the irrigation water to maintain salt treatments, and fertilized the plants biweekly by adding a quarter teaspoon of fertilizer to the water (Jack’s All Purpose 20% total N, 20% P_2_O_5_, and 20% K_2_O). After 2 months, plants were repotted into 4 L pots and we increased watering frequency to 3 times a week. Biweekly measurements included plant height, number of shoots, and salinity of the drainage and reservoir water using a portable salinity meter (YSI, Yellow Springs, Ohio). At the end of the experiment, we measured chlorophyll fluorescence as an indicator of stress using a PAM-2100 Chlorophyll Fluorometer (Walz, Effeltrich Germany) on the second collared leaf of two stems per pot. We recorded the quantum yield (*Y*) during the day, and the maximum quantum yield (*F*_v_/*F*_m_) at night. After 4 months of plant–fungal symbiosis plants were harvested and we recorded leaf number, leaf area (LI-COR LI-3100), total above and belowground dry biomass, number of shoots, lateral root length and density, and rhizome diameter. Total nitrogen and total carbon of leaf tissue was analyzed by combustion using a LECO CN628 analyzer (LECO, St. Joseph, MI, USA).

### Data analyses

We used R v.1.0.153 [51] for all data analysis and figure drawings. Paired end sequences from Illumina were processed using the dada2 package [52] and taxonomy assigned using the UNITE database [53]. The phyloseq [54] and vegan [55] packages were then used for data analysis and ggplot2 [56] for plotting figures. Samples were rarefied to 14,705 sequences which provided overall good coverage based on rarefaction curves (Supplementary Fig. 1). Samples were then filtered based on abundance, and taxa with a prevalence of at least 7% were retained. Nonmetric multidimensional scaling (NMDS) based on a Bray–Curtis dissimilarity matrix was used to visualize endophyte community composition across sites and between lineages. Permutational multivariate ANOVA (PerMANOVA) was used to assess differences between communities, and homogeneity of group dispersion was checked using the vegan functions betadisper and permutest. When PerMANOVA was significant, we used pairwise comparisons to contrast the specific factors using the package RVAideMemoire [57]. Alpha diversity based on log-transformed observed and Fisher’s alpha index was evaluated using ANOVA (type III SS). Differential abundance of taxa between lineages and across sites was evaluated using the R packages Deseq2 [58] and mvabund [59].

Differences in DSE root colonization across dates and between sites for each lineages were assessed using two-way ANOVA (type III SS) and Tukey’s post hoc means comparisons test. Pearson correlation coefficients were calculated to determine the relationship of percent colonization with sampling date and salinity.

Greenhouse results were first analyzed using ANOVA (type III SS) to evaluate if initial height was a significant explanatory variable for each parameter. When it was, the data were analyzed as an ANCOVA using covariate-adjusted means with the package emmeans [60]. Planned pairwise contrasts with a Tukey adjustment were used to assess differences between endophyte treatments at each salinity level. When the initial height was not significant, it was removed from the model and data were analyzed as an ANOVA (type III SS). Variables were log-transformed to meet ANOVA assumptions when necessary.

## Results

### Site characteristics

In site A, where pH tended to be lower than at sites B and C, percent carbon (%C) and nitrogen (%N) were higher. There were also a few site specific differences between native and invasive stands, specifically the native stand soils had higher %C in sites B and C and overall higher %N at all sites; and invasive stands had higher %SOM in sites B and C. Root morphological characteristics did not differ significantly overall between lineages or across sites (Table 1).

**Table 1:** Site and root morphology characteristics of native and invasive *Phragmites* stands. Different letters indicate significant mean differences (*p* < 0.05).

Site and *P. australis* lineage	Site A		Site B		Site C	
	Invasive	Native	Invasive	Native	Invasive	Native
Average salinity (ppt)	0.7 ± 0.2	0.7 ± 0.2	1.2 ± 0.2	1.2 ± 0.2	3 ± 0.6	3 ± 0.6
pH	6bc	5.7c	6.5a	6.3ab	6.5a	6.7a
%SOM	32.9a	31.1a	20.8b	15.9d	25.7c	21.0b
%C	16.7a	16.5a	7.9b	10.6c	10.9c	12.9d
%N	1.2a	1.3b	0.6c	0.8d	0.8e	0.9 f
Lateral root density	29.7a	13.7a	29.3a	21a	18a	27.5a
Lateral root length (cm)	5.4ab	5.9ab	4.3b	6.1a	4.1b	5.2ab
Root hair density	45.8a	9.1b	24.3b	25.3b	24.3b	13.2b

### Fungal root endophyte community analyses

**Fig. 2:**
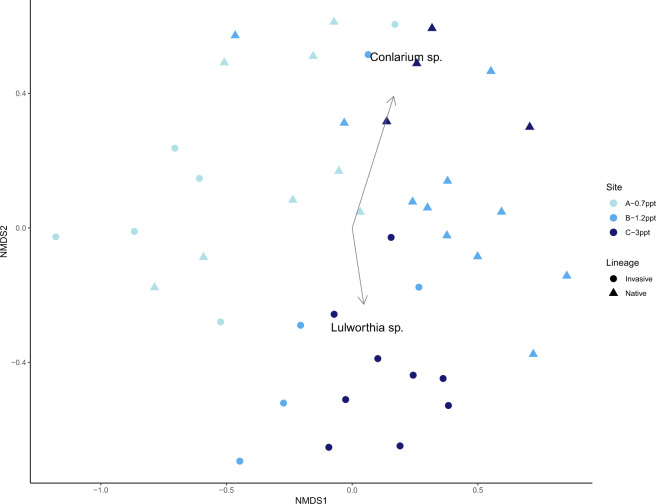
Fungal root endophyte communities differed between *P. australis* lineages and across a salinity gradient. Nonmetric multidimensional scaling plot of fungal root endophyte communities associated with native and invasive lineages of *Phragmites australis* across a salinity gradient.

After rarefaction and filtering of Illumina sequences there was a total of 165 amplicon sequence variants (ASVs). Only 83 (50%) of the ASVs could be assigned to the Genus level, and 55 (33.5%) to Species. Most fungal ASVs were present in both lineages (71%) and half of them were found at the three sites. The most abundant Orders were Lulworthiales, Agaricales, Pezizales, and Pleosporales (Supplementary Fig. 2), all of which contain taxa that have been identified as DSE [37, 61, 62]. The most abundant Genera within those orders were *Lulworthia*, *Psathyrella*, *Conlarium*, and *Anguillospora* respectively. Fungal endophytes communities did not differ between June and August (PerMANOVA, *F*_41_ = 0.99, *p* = 0.46), and were therefore combined for the rest of the analyses. When evaluating beta diversity between the sites and lineages, we found a significant interaction between factors (PerMANOVA, *F*_37_ = 2.2, *p* = 0.001). We then ran separate PerMANOVAs for each lineage and site to evaluate changes in community composition across the salinity gradient. Fungal endophytes associated with native *P. australis* in Site A (~0.7 ppt), differed from those at sites B (~1.2 ppt), and C (~3 ppt) (PerMANOVA, *F*_20_ = 2.49, *p* = 0.002). For invasive *P. australis*, fungal communities only differed between Site A and Site C (PerMANOVA, *F*_17_ = 2.79, *p* = 0.002). Contiguous stands of native and invasive *P. australis* had distinct endophyte communities in every site (Site A: *F*_12_ = 2.49, *p* = 0.01; Site B: *F*_14_ = 2.69, *p* = 0.007; Site C: *F*_11_ = 2.69, *p* = 0.02) (Fig. 2 NMDS). Alpha diversity did not differ between lineages or across sites (*p* > 0.05) (Supplementary Fig. 3). Differential taxa analysis between lineages revealed that only two of the seven differentially abundant fungi could be identified at the Genus level (Fig. 2), two others belonged to the Class Sordariomycetes and the rest could not be resolved beyond the Phylum level (two were Ascomycota and one Basidiomycota).

### DSE colonization

**Fig. 3:**
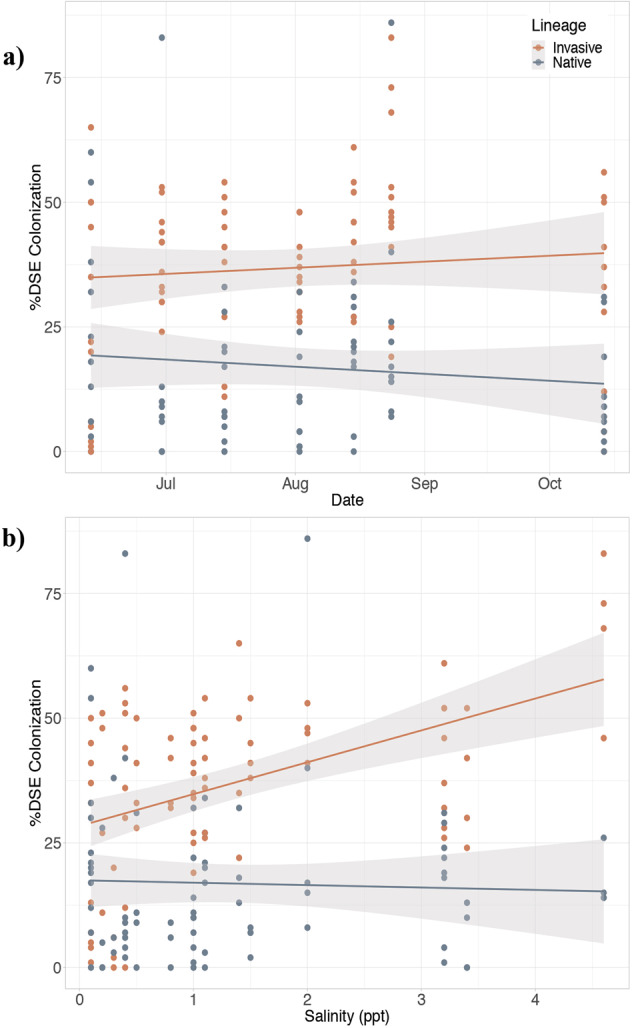
DSE colonization over a growing season and across a salinity gradient. Percent dark septate endophyte (DSE) colonization of native and invasive lineages of *Phragmites australis* (**a**) across the growing season and **b** at increasing levels of salinity.

**Fig. 4:**
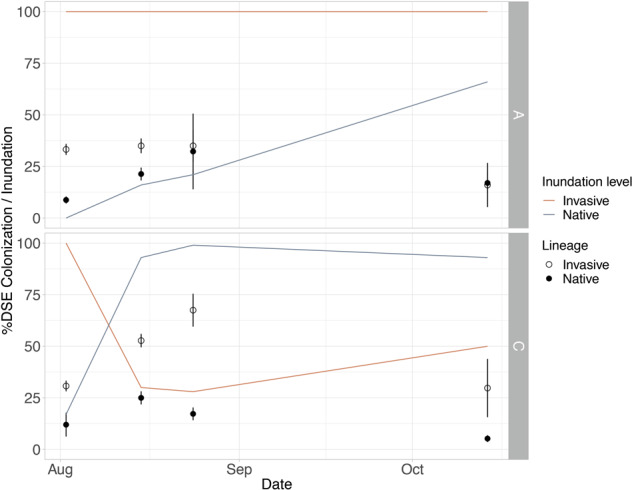
Percent dark septate endophyte (DSE) colonization and percent inundation over time at Site A (~0.7 ppt) and Site C (~3 ppt). The *y*-axis shows either the %DSE Colonization or the % inundation (percentage of time when the water was above the soil surface in the 14 days prior to sampling). The lines indicate the % inundation over time in loggers placed in Sites A (top) and C (bottom) in native and invasive *Phragmites* stands. The circles show the mean %DSE colonization for each lineage at the different sampling times, and the error bars show the standard error of the mean.

Percent DSE colonization was consistent overall throughout the growing season, and root colonization was significantly higher in the invasive than in the native lineage (*F*_147_ = 61.49, *p* < 0.01; Fig. 3a). There was a significant positive correlation between DSE colonization and salinity in the invasive lineage (*r* = 0.47, *n* = 82, *p* < 0.01), but no correlation in the native lineage (*r* = −0.037, *n* = 79, *p* = 0.75) (Fig. 3b). Inundation level had no apparent effect on the observed percent DSE colonization (Fig. 4).

### Endophyte isolation and Sanger sequencing

We isolated 15 fungal endophytes from invasive *P. australis* roots, which were categorized as DSE based on microscopic observation of DSE hyphae and microsclerotia in inoculated plant roots. Sequencing output based on the ITS region resulted in 12 contigs that predominantly matched to uncultured fungi (Supplementary Table 1). Based on ITS, only two of the isolates found matches to known cultured fungi—*Phialocephala sp*. (97.2% match) and *Trematosphaeria hydrela* (98.1%). Sequencing of the alpha-EF gene did not provide enough resolution for further differentiation of the isolates.

### Endophyte salinity tolerance

Fourteen of the isolated endophytes showed growth on PDA with up to 600 mM NaCl (Supplementary Fig. 4); therefore, all of these were tested in Magenta box laboratory assays to evaluate their effect on seedling survival of both lineages. Seedlings of invasive and native *P. australis* inoculated with endophyte GG2D showed the highest survival relative to the control treatment and other endophytes tested (Table 2). Based on these results, endophyte GG2D was selected to further evaluate its effect on salt tolerance of *P. australis* in a greenhouse experiment. Taxonomic information on this fungi could not be retrieved from BLAST but it was found to closely match a root endophyte isolated from *Persicaria amphibia* which is also an aquatic macrophyte (Supplementary Table 1). When grown on PDA, the colony was round and greyish, and grew slowly reaching a diameter of 5.5 cm after 3 weeks at 22 °C. The sterile hyphae measured about 4–7 μm wide and were mostly pigmented, but hyaline hyphae were also observed (Supplementary Fig. 4).

**Table 2:** Percent surviving seedlings in Control (non-inoculated) and DSE inoculated treatments for each lineage of *Phragmites australis* growing in MS media with 100 mM NaCl.

Endophyte	Invasive	Native
Control	50% (2/4)	13% (1/8)
**GG2D**	**100%** (**4/4)**	**86% (6/7)**
GG1E	67% (2/3)	25% (2/8)
GN	33% (1/3)	0% (0/3)
GG4B	67% (2/3)	67% (2/3)
GGI9	0% (0/4)	40% (2/5)
GG7A	33% (1/3)	50% (2/4)
GGID	67% (2/3)	50% (3/6)
GG3	25% (1/4)	NA
GG8	33% (1/3)	NA
BN3	0% (0/3)	NA
GG2C	33% (1/3)	NA
GG9	33% (1/3)	NA
GG2	0% (0/3)	NA

Numbers in parenthesis indicate the number of surviving seedlings over the total seedlings tested. For the invasive assay seedlings were added to four Magenta boxes, but some of the boxes were excluded due to contamination. For the native assay total numbers vary based on germination success, and NA indicates no seeds germinated after inoculation with that specific endophyte. Endophyte GG2D (bolded) was the selected endophyte for the greenhouse assay.

### Greenhouse assay

**Fig. 5:**
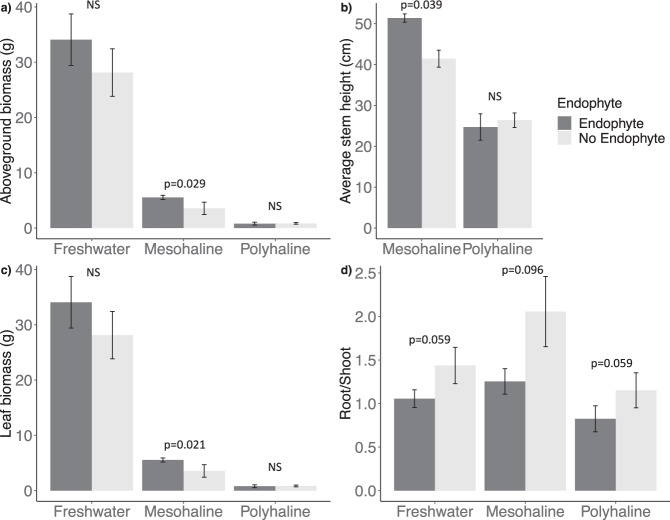
Effects of DSE inoculation on invasive *P. australis* across varying salinity levels. Bar plots showing means and standard error of the mean for the effects of dark septate endophyte inoculation of invasive *P. australis* at different salinity levels (Freshwater: no added NaCl, Mesohaline: 200 mM NaCl, and Polyhaline: 400 mM NaCl) on (**a**) aboveground biomass, (**b**) average stem height, (**c**), leaf biomass, and (**d**) root:shoot ratio. NS indicates *p* values greater than *p* = 0.1. Average stem height for freshwater treatments was not recorded because there were sometimes over one hundred per pot making it difficult to get an accurate count.

Endophyte inoculation increased aboveground biomass of invasive *P. australis* only at the mesohaline salinity treatment (ANCOVA, contrast *t*_15_ = 2.42, *p* = 0.029) (Fig. 5a). This was mainly driven by a significant increase in average stem height (ANOVA, *F*_11_ = 6.77, *p* = 0.039) (Fig. 5b) and leaf biomass (ANCOVA, contrast *t*_15_ = 2.58, *p* = 0.021) (Fig. 5c), and an increase in stem biomass (ANCOVA, contrast *t*_15_ = 2.1, *p* = 0.053) at that salinity level in inoculated plants. Other aboveground parameters, including number of stems, leaf count, and leaf area, did not differ significantly between inoculated and non-inoculated plants (Supplementary Table 2). There was also no effect of inoculation on belowground parameters including rhizome and lateral root biomass, rhizome diameter, and lateral root length and number (*p* > 0.05, Supplementary Table 2); but overall root-to-shoot ratio was lower in endophyte treatments across all salinity levels (*p* < 0.1) (Fig. 5d).

We also did not observe endophyte or salinity treatment effects on some of the other measured variables. Photosynthetic efficiency based on quantum yield (*Y*) and maximum quantum efficiency (*F*_v_/*F*_m_) did not differ between salinity treatments (ANOVA, *Y*: *F*_16_ = 1.3, *p* = 0.29; *F*_v_/*F*_m_: *F*_16_ = 0.37, *p* = 0.69), or between inoculated and not inoculated plants (ANOVA, *Y*: *F*_16_ = 0.1, *p* = 0.75; *F*_v_/*F*_m_: *F*_16_ = 1.02, *p* = 0.33) (Supplementary Table 2). Similarly, total carbon content in leaf tissue was not affected by salinity or fungal treatments (ANOVA, *F*_15_ = 0.28, *p* = 0.75; *F*_15_ = 0.08, *p* = 0.78; respectively) and total nitrogen did not differ between inoculated and non-inoculated plants (*F*_15_ = 0.64, *p* = 0.44). However, salinity did affect total leaf nitrogen content (ANOVA, *F*_15_ = 8.73, *p* = 0.003), and was highest in mesohaline conditions and lowest in freshwater (Supplementary Table 2).

## Discussion

Native and invasive lineages of the common reed *P. australis* are colonized by distinct fungal endophytes that can improve the salt tolerance of these grasses. We observed an increase in DSE colonization with salinity in the invasive lineage, and greater overall colonization of this lineage relative to the native (Fig. 3a); we speculated this was due to a mutualistic association between DSE and the invasive lineage, likely related to salt tolerance. This warranted further investigation on the potential role of DSE in salinity tolerance of invasive *P. australis*, which has not been considered a relevant factor to explain *P. australis* expansion into saline areas so far.

The “habitat adapted hypothesis” [8] suggests that plants may associate with endophytes to improve their tolerance to environmental stress, and these endophytes can confer similar stress tolerance to genetically distant plants. This has been commonly reported for Class II endophytes that can benefit a host under a specific stress, and induce a similar response in closely related hosts [1, 8, 63]. Similarly, in our study Class IV DSE isolated from roots of invasive *P. australis* improved salt tolerance of both the invasive and native lineage (Table 2). This suggests that DSE mutualisms may be an additional mechanism of salt tolerance for *P. australis* that might enhance the invasion of the European lineage. On the other hand, these mutualisms could also be useful in restoration of the native lineage if inoculation improves its survival in areas susceptible to saltwater intrusions [28].

DSE associations can range from parasitic to mutualistic, but are predicted to be primarily the latter in plants under abiotic stress [64]. Accordingly, our greenhouse study showed that invasive *P. australis* did not appear to benefit from inoculation under freshwater conditions; but had higher aboveground biomass at mesohaline salinity (Fig. 5). These results highlight the importance of environmental drivers of plant–fungal symbiosis, as has been seen with mycorrhizae and other endophytic fungi [1, 65]; and can help explain why there are such mixed results in the literature concerning the effects of DSE on their hosts [66, 67].

An increase in photosynthetic efficiency is one of the mechanisms by which DSE could enhance plant tolerance to abiotic stress [68, 69]. In our study DSE inoculation had no effect on quantum yield (*Y*) or maximum quantum yield (*F*_v_/*F*_m_), and did not affect C or N content in leaves; so growth promotion was likely related to other factors. For example the reduction of reactive oxygen species by fungal melanins could promote plant tolerance to various types of abiotic stress including high salinity [70]; and melanin isolated from a DSE was reported to have high antioxidant activity [71]. Endophyte colonization could also affect plant–water relationships and promote salt tolerance through other mechanisms. In the mycorrhizal fungal symbiosis hyphae can directly uptake water into the plant, induce changes in gene expression relevant to osmotic stress, and increase the production of osmolytes [72]. Although these mechanisms of salt tolerance have not been specifically studied in DSE yet, production of trehalose and mannitol has been reported in these endophytes [2] and accumulation of these osmolytes could reduce salinity stress in colonized plants [70].

Fungal inoculation led to a marginally significant decrease in root:shoot ratio relative to non-inoculated controls (Fig. 5), as inoculated plants generally favored above over belowground growth. This is often observed in mycorrhizal plants where extensive hyphal networks promote water and nutrient uptake [73, 74], but a meta-analysis on DSE found no influence of inoculation on plant root:shoot ratio, and an overall increase in both root and shoot biomass [66]. We only identified the latter in our greenhouse assay and suggest that preferential allocation of C aboveground was not a result of an imposed abiotic stress, but rather a response of the host to inoculation.

In eutrophic wetlands, like our study system, greater aboveground biomass could translate to a significant competitive advantage. Given the abundance of nitrogen [75] and phosphorus in these wetlands [76], belowground competition for these resources is relaxed, and aboveground competition might have a greater role in plant community structure [77, 78]. Other factors to consider would be soil characteristics, disturbance, and the plant’s own physiological adaptations which can be key to determine the outcome of plant competition and contribute to plant invasions in wetlands [20, 28]. Based on our greenhouse findings, we propose DSE could play a role in expansion and establishment of invasive *P. australis* into brackish marshes by increasing its competitive ability.

Characterizing microbial communities of the native and invasive lineages, and identifying relevant microbial associations can help improve management of *P. australis* [14, 15]. Our study characterized fungal endophyte communities of contiguous stands of native and invasive *P. australis* and showed that community composition was lineage and site specific (Fig. 2), even though most taxa were present in both lineages and half of them were found at all sites. These results differ from those reported by Bickford et al. [79] who found no differences in root fungal endophytes between *P. australis* lineages in the Great Lakes, USA. However, soil saturation was a relevant environmental factor in that study, whereas water level did not appear to play a role in endophyte colonization or community structure in our study sites (Fig. 4). Differential abundance analysis showed that the genera *Conlarium* and *Lulworthia* were associated with native and invasive *Phragmites* respectively (Fig. 2). *Conlarium* sp. are DSE that have been found to be symbiotic in sugarcane roots [80] and *Lulworthia* sp. are DSE commonly isolated from marine and coastal systems where they are known to associate with roots of sea grasses and salt marsh plant species [62, 81]; the ecological roles of these endophytes are still unknown.

Dark septate endophyte colonization has been reported to vary seasonally, showing a decrease at the end of the growing season in alpine plant communities and in a tall grass prairie [82, 83]. In our study DSE colonization was prevalent in both lineages throughout the growing season (Fig. 3b), and likely underestimated because hyaline hyphae, which are harder to detect with our staining method, were not quantified [40]. The high prevalence of DSE and lack of evident disease symptoms in colonized plants, suggest a relevant and yet unexplored, role of these endophytes in our study system even at low salinities.

Our study focused on the effects of DSE on salt tolerance, and specifically looked at NaCl as a stress factor; future research could further address the ecological role of DSE in wetland plants by looking at their effects on sulfide tolerance for example. This would be particularly relevant in freshwater wetlands where saltwater intrusion is already affecting coastal biogeochemistry and plant community composition [84, 85]. Increased sulfide concentrations can be toxic for aquatic macrophytes and can limit their growth by reducing nitrogen uptake [86]. Given that DSE might help plants incorporate nitrogen [45, 66], it would be interesting to know if they can improve *P. australis’* tolerance to sulfide toxicity. Sulfide could also have a negative effect on seedlings that colonize sites after invasive *P. australis* removal [87], so beneficial effects for plant restoration using DSE should also be evaluated. In conclusion, *P. australis* could benefit from DSE colonization when exposed to salt stress. Therefore, the role of fungal mutualists, particularly in a context of sea-level rise, is worth considering in future studies of invasion ecology, species management, and restoration of native plants.

## Supplementary information

Supplementary Figure 1

Supplementary Figure 2

Supplementary Figure 3

Supplementary Figure 4

Supplementary Table 1

Supplementary Table 2
