# Supplementary material for: Dark septate endophyte improves salt tolerance of native and invasive lineages of Phragmites australis
Source: ISME J. 2020 Apr 27;14(8):1943–54. doi: 10.1038/s41396-020-0654-y (PMC7367851; doi:10.1038/s41396-020-0654-y)
Supplement: Supplementary file 1 — Supplementary Figure 1 [file 41396_2020_654_MOESM1_ESM.docx]

**Supplementary Figure 1**

**Fig. S1:** Rarefaction curves for ITS Illumina sequences showing cutoff at 14,705 sequences.
