# Supplementary material for: Dark septate endophyte improves salt tolerance of native and invasive lineages of Phragmites australis
Source: ISME J. 2020 Apr 27;14(8):1943–54. doi: 10.1038/s41396-020-0654-y (PMC7367851; doi:10.1038/s41396-020-0654-y)
Supplement: Supplementary file 2 — Supplementary Figure 2 [file 41396_2020_654_MOESM2_ESM.docx]

**Supplementary Figure 2**

**Fig. S2:** Relative abundance of the ten most abundant orders (a) and genera (b) of fungal root endophytes found in native and invasive lineages of *Phragmites australis*.
