# Supplementary material for: Dark septate endophyte improves salt tolerance of native and invasive lineages of Phragmites australis
Source: ISME J. 2020 Apr 27;14(8):1943–54. doi: 10.1038/s41396-020-0654-y (PMC7367851; doi:10.1038/s41396-020-0654-y)
Supplement: Supplementary file 3 — Supplementary Figure 3 [file 41396_2020_654_MOESM3_ESM.docx]

**Supplementary Figure 3**

**Fig. S3** Alpha diversity of fungal root endophytes of native and invasive *Phragmites australis* across a salinity gradient (A=~0.7 ppt, B=~1.2 ppt and C=~3 ppt). Diversity was measured at each stand and is based on Illumina sequencing results.
