# Supplementary material for: Dark septate endophyte improves salt tolerance of native and invasive lineages of Phragmites australis
Source: ISME J. 2020 Apr 27;14(8):1943–54. doi: 10.1038/s41396-020-0654-y (PMC7367851; doi:10.1038/s41396-020-0654-y)

**Supplementary Figure 4**

**Fig S.4:** a) Dark septate endophyte hyphae and microsclerotia colonizing root of invasive *Phragmites australis*. b) Hyphae of dark septate endophyte (GG2D) used in greenhouse experiment. c) Dark septate endophyte isolates grown in PDA+600mM NaCl after 6 weeks. Endophyte GG2D is shown on the bottom left plate. Scale bar=70μm

**c**


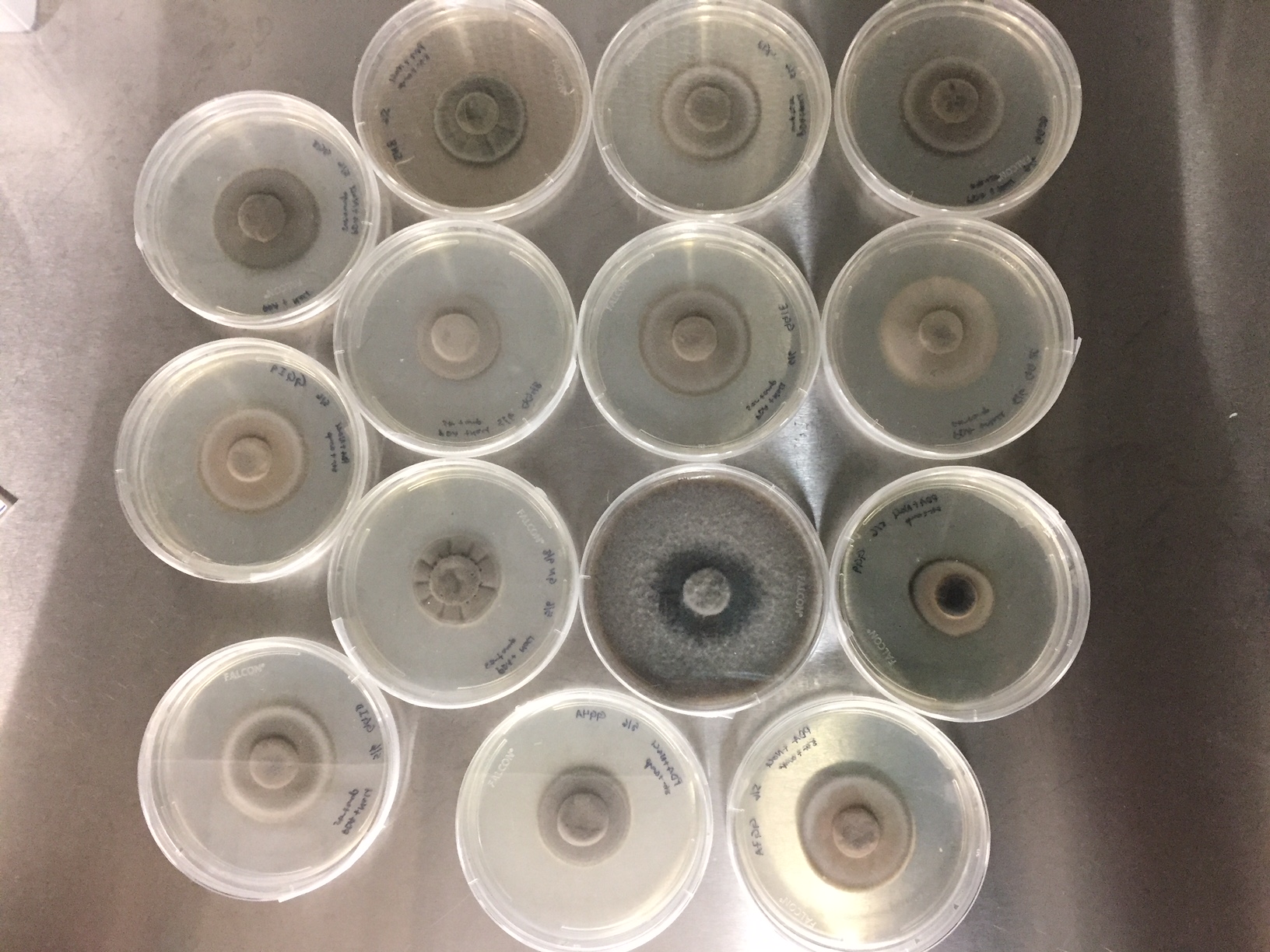

Supplement: Supplementary file 4 — Supplementary Figure 4 [file 41396_2020_654_MOESM4_ESM.docx]
