# Supplementary material for: Dark septate endophyte improves salt tolerance of native and invasive lineages of Phragmites australis
Source: ISME J. 2020 Apr 27;14(8):1943–54. doi: 10.1038/s41396-020-0654-y (PMC7367851; doi:10.1038/s41396-020-0654-y)
Supplement: Supplementary file 5 — Supplementary Table 1 [file 41396_2020_654_MOESM5_ESM.docx]

**Supplementary table 1:** Description of BLAST results for Sanger sequences of fungal root endophytes isolated from invasive *Phragmites australis*.

| Endophyte | % Similarity and Closest BLAST match based on ITS region | Characterization | Reference |
| --- | --- | --- | --- |
| GG2D | 97%: Fungal endophyte voucher ARIZ:DM0192 18S ribosomal RNA gene and internal transcribed spacer 1, partial sequence | Fungal endophyte isolated from healthy, mature submerged root of *Persicaria amphibia* (swamp smartweed) in Willow Creek Reservoir, Arizona  Taxonomy: NA | Sandberg DC, Battista LJ, Arnold AE. Fungal endophytes of aquatic macrophytes: diverse host-generalists characterized by tissue preferences and geographic structure. Microb Ecol. 2014;67(4):735–747.  Accession number: KF673730.1 |
| GGIE, GG4B, GGI9, GGID, GG8, BN3, GG2C, GG4A, GG10 | 87.4%-93.1%: Fungal sp. strain S184S internal transcribed spacer 1, partial sequence; 5.8S ribosomal RNA gene and internal transcribed spacer 2, complete sequence; and 28S ribosomal RNA gene, partial sequence | Cultured fungus isolated from *Ammophila breviligulata* (American beachgrass)  Taxonomy: NA | David, AS, Seabloom, EW, May, G. Disentangling environmental and host sources of fungal endophyte communities in an experimental beachgrass study. Mol Ecol. 2017; 26: 6157– 6169  Accession number: KU839097.1 |
| GG7A | 96%: Fungal sp. 51 SAB-2015 strain SV664 internal transcribed spacer 1, partial sequence; 5.8S ribosomal RNA gene and internal transcribed  spacer 2, complete sequence; and 28S ribosomal RNA gene, partial  sequence. | Isolated from *Spartina alterniflora* (smooth cordgrass) in Barataria Bay, USA  Taxonomy: NA | Kandalepas D, Blum MJ, Van Bael SA (2015) Shifts in Symbiotic Endophyte Communities of a Foundational Salt Marsh Grass following Oil Exposure from the Deepwater Horizon Oil Spill. PLoS ONE 10(4): e0122378.  Accession number: KP757570.1 |
| GG9 | 98.1%: *Trematosphaeria hydrela* genomic DNA sequence contains ITS1, 5.8S rRNA gene and ITS2, isolate F259 | Isolated from *Arabis alpina* (alpine rock-cress) surface sterilized roots  Taxonomy: Eukaryota; Fungi; Dikarya; Ascomycota; Pezizomycotina; Dothideomycetes; Pleosporomycetidae; Pleosporales; Massarineae; Trematosphaeriaceae; Trematosphaeria | Almario, Juliana & Jeena, Ganga & Wunder, Jörg & Langen, Gregor & Zuccaro, Alga & Coupland, George & Bucher, Marcel. (2017). Root-associated fungal microbiota of nonmycorrhizal *Arabis alpina* and its contribution to plant phosphorus nutrition. Proceedings of the National Academy of Sciences. 114.  Accession number: LT821517.1 |
| GG3 | 97.2%: Cf. *Phialocephala sp*. AU_BD15 internal transcribed spacer 1, partial sequence; 5.8S ribosomal RNA gene, complete sequence; and internal transcribed spacer 2, partial sequence | Taxonomy: Eukaryota; Fungi; Dikarya; Ascomycota; Pezizomycotina; Leotiomycetes; Helotiales; Helotiales incertae sedis; Phialocephala. | *Phialocephala sp*. strain AU_BD15  Culture collection: Gareth Griffith, Wales, UK  Accession number: JN995646.1 |
| GN, GG2 | NA | Excluded from analysis due to poor sequencing quality |  |
