# Supplementary material for: Dark septate endophyte improves salt tolerance of native and invasive lineages of Phragmites australis
Source: ISME J. 2020 Apr 27;14(8):1943–54. doi: 10.1038/s41396-020-0654-y (PMC7367851; doi:10.1038/s41396-020-0654-y)
Supplement: Supplementary file 6 — Supplementary Table 2 [file 41396_2020_654_MOESM6_ESM.docx]

**Supplementary table 2:** Mean and standard error of (a) aboveground and (b) belowground parameters, (c) photosynthetic efficiency and (d) total carbon and nitrogen. Leaf count was not recorded for freshwater treatments due to high number of stems. N=4

**a)**

| Greenhouse Treatment | | Stem number | Leaf count | Leaf area | Stem biomass |
| --- | --- | --- | --- | --- | --- |
| Salinity | Endophyte |  |  |  |  |
| Freshwater | Inoculated | 87.3 ± 12.2 | NA | 4652.7 ± 569.8 | 36.17 ± 4.0 |
| Freshwater | No Endophyte | 66.8 ± 10.9 | NA | 4274.1 ± 641.7 | 35.88 ± 3.3 |
| Mesohaline | Inoculated | 21.0 ± 2.0 | 153.3 ± 12.8 | 1053.8 ± 103.1 | 7.4 ± 1.2 |
| Mesohaline | No Endophyte | 17.0 ± 1.0 | 145 ± 13.7 | 744.14 ± 145.2 | 4.5 ±0.5 |
| Polyhaline | Inoculated | 5.8 ± 0.8 | 30.5 ± 13.7 | 79.9 ± 33.7 | 1.2 ± 0.3 |
| Polyhaline | No Endophyte | 5.8 ± 1.1 | 39.3 ± 6.3 | 81.1 ± 13.7 | 1.1 ± 0.2 |

**b)**

| Greenhouse Treatment | | Rhizome biomass | Rhizome diameter | Lateral root biomass | Lateral root number | Lateral root length |
| --- | --- | --- | --- | --- | --- | --- |
| Salinity | Endophyte |  |  |  |  |  |
| Freshwater | Inoculated | 50.3 ± 8.2 | 4.2 ± 0.3 | 25.5 ± 9.0 | 7.8 ± 1.5 | 4.4 ± 1.0 |
| Freshwater | No Endophyte | 51.7 ± 11.7 | 4.6 ± 0.1 | 42.8 ± 15.5 | 8.5 ±1.1 | 4.5 ± 0.3 |
| Mesohaline | Inoculated | 12.1 ± 1.0 | 4.2 ± 0.3 | 3.7 ± 0.7 | 10.6 ± 1.1 | 9.5 ±0.8 |
| Mesohaline | No Endophyte | 11.9 ± 0.9 | 3.2 ± 0.1 | 3.8 ± 0.6 | 9.1 ± 1.6 | 9.0 ± 1.3 |
| Polyhaline | Inoculated | 1.2 ± 0.6 | 3.4 ± 0.02 | 0.8 ± 0.3 | 8.5 ±1.1 | 10.2 ± 0.8 |
| Polyhaline | No Endophyte | 1.8 ± 0.6 | 2.7 ± 0.2 | 0.7 ± 0.2 | 8.4 ± 0.8 | 10.1 ± 2.6 |

**c)**

| Greenhouse Treatment | | Yield | Fv/Fm |
| --- | --- | --- | --- |
| Salinity | Endophyte |  |  |
| Freshwater | Inoculated | 0.699 ± 0.015 | 0.784 ± 0.018 |
| Freshwater | No Endophyte | 0.705 ± 0.012 | 0.806 ± 0.010 |
| Mesohaline | Inoculated | 0.714 ± 0.007 | 0.794 ± 0.019 |
| Mesohaline | No Endophyte | 0.707 ± 0.007 | 0.819 ± 0.005 |
| Polyhaline | Inoculated | 0.686 ± 0.015 | 0.803 ± 0.022 |
| Polyhaline | No Endophyte | 0.727 ± 0.013 | 0.831 ± 0.003 |

**d)**

| Greenhouse Treatment | | %C | %N |
| --- | --- | --- | --- |
| Salinity | Endophyte |  |  |
| Freshwater | Inoculated | 41.4 ± 0.4 | 2.77 ± 0.1 |
| Freshwater | No Endophyte | 41.3 ± 0.3 | 2.62 ± 0.1 |
| Mesohaline | Inoculated | 41.8 ± 0.2 | 3.59 ± 0.1 |
| Mesohaline | No Endophyte | 41.8 ± 0.3 | 3.66 ± 0.03 |
| Polyhaline | Inoculated | 41.8 ± 0.8 | 3.19 ± 0.3 |
| Polyhaline | No Endophyte | 42.14 ± 0.17 | 3.39 ± 0.1 |
